# News and Events of the Week

**Published:** 1910-10-15

**Authors:** 


### News and Events of the Week.

At the Central London Throat and Ear Hospital, Gray's Inn Road, the following lectures will take place: On Tuesday, October 18, at 3.45 p.m., "Pharynx and Naso-Pharynx," by Dr. Atkinson; and on Friday, October 21, at 3.45 p.m., "Clinical Cases," by Dr. Abercrombie.

The annual series of six demonstrations of specimens in the museum of the Royal College of Surgeons will be given in the theatre of the college by Professor Arthur Keith and Professor Samuel G. Shattock on each Friday and Monday afternoon, beginning on October 21.

Dr. David Wilson Hogue, M.D., D.D.S., of Marlborough Road, Bournemouth, formerly of the Edinburgh Dental Dispensary (afterwards the Edinburgh Dental Hospital), and formerly examiner for the dental diploma of the Royal College of Surgeons, who died on August 5, has left, in addition to real estate, personal estate in the United Kingdom valued at £59,367.

The fourth centenary of the birth of Dr. John Caius, the great second founder of Gonville and Caius College, Cambridge, was celebrated last week. Sir Thomas Barlow spoke at the celebration, and brought with him the ancient caduceus from the College of Physicians to lie beside that given by Caius himself. Dr. John Venn, the learned historian of the College, delivered an "In Memoriam" address, and the occasion was marked by the Latin telegram of congratulation which was received from the University of Padua.

The presidential address of the Medical Society of London at the opening of the session was delivered by Mr. Charters J. Symonds, M.D., M.S., F.R.C.S., on "The Treatment of Chronic Abscess of the Bone by Means of Metal Drains," on Monday last. The address was illustrated by patients, preparations, and radiograms. A discussion was opened by Sir W. Watson Cheyne and Mr. A. E. Barker.

The following awards of entrance scholarships and exhibitions to the Faculty of Medical Sciences at University College, London, have been made: The Bucknill Scholarship and First Medical Exhibition of the value of 190 guineas, divided equally between Mr. W. A. E. Karunaratne and Mr. C. J. H. Sharp. The Second Medical Exhibition, of the value of 55 guineas, to Mr. H. A. Fawcett; *Proxime Accessit*, Mr. E. A. L. Sansom. The Epsom Scholarship to Mr. H. L. G. Hughes.

Muriel Lady Helmsley named recently, in the gardens of the Royal Botanic Society at Regent's Park, the second caravan built for the Women's Imperial Health Association of Great Britain, of which she is president. The new caravan, named the "Florence Nightingale," has started on a tour of the Eastern counties, beginning at Romford. Dr. H. J. F. Simson, who presided at the inaugural ceremony, stated that lectures would deal with such subjects as "How to dust a room," "How to feed a baby," and "The effects of improperly feeding children." A third caravan, "The Aurora," which was also on view in the gardens, has started on a tour in the London parks.

Dr. J. R. Kaye, the medical officer of the West Riding of Yorkshire, has been presented by the local branch of the Incorporated Society of Medical Officers (of which he has been Secretary and Treasurer for seventeen years) with a handsome silver centrepiece on the occasion of his silver-wedding.

A special resolution of the Sanitary Committee of the Brighton Corporation records its appreciation of the valuable services rendered to the town by Dr. Duncan Forbes, M.O.H., as Honorary Secretary of the local committee entrusted with the arrangements for the successful Congress of the Royal Sanitary Institute.

Mr. F. Gowland Hopkins, M.B., F.R.S., late natural science tutor at Emmanuel College, and formerly demonstrator in physiology at Guy's Hospital, has been admitted to a Fellowship at Trinity College, Cambridge, upon receiving the appointment of prælector in bio-chemistry. This appointment is to be held conjointly with the University Readership in Chemical Physiology previously held by him.

Dr. Walter Strover, M.R.C.S., of Mintern House, Hoxton, who disappeared suddenly nearly three weeks ago after having attended to a number of patients, was last week found dead in a Bedfordshire wood. Several articles found on the body of the doctor were identified by the relatives. The body was found fully dressed, and bore no signs of violence. His brother was called to identify the body, but was unable owing to its decomposed condition to do so. The deceased was fifty-six years of age, and an old Guy's man, and was formerly medical officer of health for Chingford. He relinquished his office of medical officer and other appointments in order that he might devote the whole of his time to the poor of North London.

A meeting was held on June 23, 1910, at the Marylebone Dispensary, 77 Welbeck Street, London, W., to consider the advisability of starting a society consisting of those interested in the work of "Infant Consultations." The following resolutions were put to the meeting and carried unanimously, subject to amendment at the general meeting to be held on October 19, 1910, at 6 p.m.: (1) That a society of officers of "Infant Consultations" be and is hereby constituted; (2) and that the aim and object of such society be to (*a*) bring into closer relationship all those engaged or interested in the work of such institutions; (*b*) to promote the establishment of similar institutions and to advise as to their organisation; (*c*) from time to time to hold meetings for the reading of papers and the holding of discussions on subjects germane to the work of "Infant Consultations"; (*d*) for the recording of experience gained by individuals engaged in the work; (*e*) for the collection of literature, statistics, and reports bearing on the subject; (3) that the society consist of a president, vice-president, honorary secretaries, treasurer, librarian, and ordinary members; (4) that the expenses of the society be defrayed by an annual subscription of 2s. 6d. An Executive Committee has already been formed. All communications should be addressed to the Hon. Secretaries, Dr. Ronald Carter, 11 Leonard Place, Kensington, W.; Dr. J. Lane-Claypon, 69 Prince of Wales Mansions, Battersea Park, S.W.

The following are the arrangements for next week at the West London Postgraduate College: Demonstrations by the Surgical Registrar at 10 a.m., on October 17 and 20; Demonstration by the Medical Registrar at 10 a.m., on October 21; Demonstration of Minor Operations at 11.30 a.m., on October 18; Pathological Demonstration at 12 noon, on October 17; and Lecture by Dr. Grainger Stewart on "Practical Medicine" at 12.15 p.m. on October 19. Lectures will be given at 5 p.m. on October 17, by Mr. Dunn, "Glaucoma, Its Varieties and Treatment"; October 18 and 19, by Dr. Grainger Stewart, on "The Diagnosis of Functional and Organic Disorders of the Nervous System" (in two parts); October 20, by Mr. Baldwin, on "Practical Surgery" (Lecture 2); and October 21, by Dr. Abraham, on "Cases of Skin Diseases."

Next week's arrangements at the Medical Graduates' College and Polyclinic, 22 Chenies Street, W.C., are as follows: Monday, October 17, at 4 p.m., Dr. Colcott Fox, Clinique (Skin); at 5.15 p.m., Dr. George Oliver, "Some Bedside Clinical Methods," with Demonstrations. Tuesday, at 4 p.m., Dr. Purves Stewart, Clinique (Med.); at 5.15 p.m., Dr. E. C. Hort, "Diagnosis and Treatment of Gastric and Duodenal Ulcer" (continued). Wednesday, at 4 p.m., Mr. D. C. L. Fitzwilliams, Clinique (Surg.); at 5.15 p.m., Mr. A. W. Mayo Robson, "Certain Points in Diagnosis and Treatment arising out of his last 150 Operations for Duodenal Ulcer." Thursday, at 4 p.m., Mr. J. Jackson Clarke, Clinique (Surg.); at 5.15 p.m., Dr. J. F. Halls Dally, "Respiration in Health and Disease." Friday, at 4 p.m., Mr. Angus McNab, Clinique (Eye).

The following demonstrations of specimens in the Museum of the Royal College of Surgeons of England will be given, at 5 p.m., in the theatre of the college in Lincoln's Inn Fields in the October and November course: October 21, by Prof. Keith, Specimens illustrating obstruction of the bowel caused by (1) defects of the Diaphragm; (2) Peritoneal pockets; (3) imperfect attachment of the Mesentery; October 24, by Mr. Shattock, Specimens illustrating Syphilis; October 28, by Prof. Keith, Specimens illustrating the various forms of imperfect differentiation of the Sexual Organs and characters; October 31, by Mr. Shattock, Specimens illustrating Rickets, Cretinism, etc.; November 4, by Prof. Keith, Specimens illustrating Acromegaly and some allied conditions; and November 7, by Mr. Shattock, Specimens illustrating Injuries and Diseases of Arteries. The demonstrations are intended for advanced students and medical practitioners.

